# COVID-19 Pandemic: Hard lessons to learn

**DOI:** 10.4314/ahs.v23i1.12

**Published:** 2023-03

**Authors:** Mohamud Sheek-Hussein, Fikri M Abu-Zidan

**Affiliations:** 1 Institute of Public Health; College of Medicine and Health Sciences, United Arab Emirates University Al-Ain, United Arab Emirates; 2 Harvard University, T.H Chan School of Public Health, Boston, MA, USA; 3 Loma Linda University School of Public Health, Loma Linda, California, USA; 4 The Research Office, College of Medicine and Health Sciences, United Arab Emirates University Al-Ain, United Arab Emirates

“*That men do not learn very much from the lessons of history is the most important of all the lessons that history has to teach***”** - Aldous Huxley 1894-1963

## We were not ready

The novel Coronavirus (2019-nCoV) was first detected in Wuhan, the capital of the Hubei province in China^1^. Authorities at the World Health Organization (WHO) were informed at the end of December 2019 of a cluster of pneumonia-related illnesses of unknown etiology. The illnesses were later isolated and identified as a new type of coronavirus on January 7, 2020^2^. The initial outbreak of the virus was linked locally to a seafood animal market in Wuhan City^3^, which had disseminated quickly to distant regions. More cases were reported in mainland China as well as in the surrounding countries such as Thailand, Japan, and the Republic of Korea. On January 13, 2020, the WHO declared Covid-19 Pandemic as an international public health emergency^4^, implying that it was serious, unusual, and unexpected^5^. As of June 13, 2022 of the John Hopkins report on the Global cases of COVID-19 has shown more than 535 million confirmed COVID-19 cases, more than six million and three hundred thousand COVID-19 related deaths, and more than eleven billion and half were vaccinated.^6^

It soon became clear that the world was not properly prepared for viral pandemics; Alarmingly, as of June 4, 2022, many countries reported a novel viral monkeypox disease. Despite ongoing epidemiological investigations, around 800 laboratory-confirmed cases of monkeypox have been reported to the WHO from outside of endemic countries.^7^ Many shortages and numerous flaws in the global health system existed. Those persons without COVID-19 vaccination and health workers without proper protective personal equipment were seriously impacted. According to the WHO: “the World Health Assembly agreed to launch a process to develop a historic global accord on pandemic prevention, preparedness and response. This was an important step toward addressing the need for global cooperation to manage this unprecedented human catastrophe as recognized by the world leaders^8^. The western wealthy countries attempted to control the pandemic through non-pharmaceutical interventions and vaccination. Despite that, the vaccination rates among the high-risk group population in some European countries are still insufficient indicating a need for harmonized effort to overcome the transmission of the virus.^9^ Complete covid -19 vaccination and booster doses might limit the disease transmission in wealthy countries. However low-income countries might suffer unavailability even of the first dose.^10^

Mitigation of the COVID-19 Pandemic at its early stages aimed to reduce its negative effects on individuals and their communities. This included non-pharmaceutical interventions such as mandatory facial mask covering, emphasis on individual and social distancing, personal hygiene, education awareness, lockdown campaigns, and stay-at-home orders. A systematic review by Talic et al investigated the impact of non-pharmacological public health measures on the COVID-19 Pandemic. They showed that proper use of face masks was effective in preventing COVID-19 infection in social gatherings of more than six persons. Other useful interventions include screening of the virus, establishing surveillance for active cases, close contact tracing, self-isolation and quarantine. These mitigation measures are particularly useful for vulnerable people who have an increased risk of comorbidities.^11^

## Struggling with reality

Infectious epidemics have similar history including plague in 1346, smallpox in 1870 and Spanish influenza in 1918. More recent epidemic outbreaks include the SARS, MERS-COV, Ebola, Zika, H1N1, Swine flu, and recently monkeypox virus. COVID-19 virus has many similarities to SARS and MERS-COV viruses. They share the same etiology, RNA structure, and symptoms presentations, and are of zoonotic origin. Nevertheless, they differ in the mode of transmission, severity, and prognosis. As such, modes of prevention and control measures are different. The magnitude of the Covid-19 spread is unlike any of the past epidemics. The COVID-19 virus continues to evolve rapidly and spread beyond control regardless of available vaccination types. The unpredicted continuous mutations in the genetic code are related to a group of virus variants that were derived from a common ancestor. Multiple variants of SARS-CoV-2 (Alpha, Beta, Gamma) have been identified worldwide during the COVID-19 Pandemic.^12^ With the newest variant being the Omicron (B.1.1.529). This variant, which was of global concern, was first detected and reported to WHO from South Africa on November 24, 2021^13^, spreading to neighboring countries which were facing health disparity, inaccessibility to vaccines, and precarious socio-economic and poor unfavourable environmental conditions. These continuous emerging and re-emerging genetic mutations in developing countries pose a threat to the global public health including high-income countries. Accordingly, research on infectious disease in developing countries should be given a fair portion of the global research funds 14.

Different global governments are not genuinely collaborating to control this Pandemic. The implementation of vaccine passports and discriminatory travel bans have done little to curb the spread. In reality, some Western countries relaxed the protective control measures (because of personal freedom issues), allowing the spread of misinformation and vaccine distrust, which has in turn caused growing anti-vaccine rhetoric and vaccine hesitancy. Thus, the low rate of vaccine uptake in some European countries will possibly contribute to the occurrence of a fourth and possibly fifth wave of COVID-19 virus spread 15.

## All are living in one world

The one health and one world concept indicate that the WHO and the World Organization for Animal Health (OIE) should collaborate in implementing this concept by recognizing that the health of a nation and its people is connected to the health of the animals because they share one environment. As a result of globalization, easy movements of the people, animals, merchants, and food products encourage the fast spread of infectious epidemics across the world without barriers between borders. There was no country saved from facing the challenges of COVID-19. Western developed countries like the United Kingdom (UK) and Italy had major difficulties in managing the COVID-19 Pandemic during 2020 16. Italy was hit hard in the early stages of the pandemic with more than 400 000 confirmed cases and more than 36 000 COVID-attributed deaths (as of mid-October 2020). Paradoxically during this crisis, the former Italian colony “Of Somalia” were doing relatively well to spare their best medical doctors and send them to help the Italian community during that period^17^,^18^. This Pandemic revealed the major shortages present in the global health care systems; even in the most advanced Western countries, contrary to their resilient image. They failed to digest the lessons learned from the previous epidemics like SARS, MERS, and Ebola. This was reflected in the poor coordination, poor emergency preparedness, and poor response during the initial stage of the Pandemic 19 16, lacking the capacity to manage the critically-ill COVID-19 patients and to protect the frontline health care workers. In contrast, some African countries, despite their limited resources, have shown proper resilience towards the Pandemic by recognizing its public health urgent needs^19^.

## Scientific integrity and ethics

The current pandemic has generated an exponential research outcome on COVID-19. PubMed search on June 14, 2022 has shown more a quarter million articles on this topic generated in the last two years 20. Despite this, misinformation from non-reliable sources on the virus, its origin, vaccine efficacy, and different screening tests affected the public opinion. The misinformation has potentially a long-term social influence on the behaviour among individuals, in the understanding of the problem, and their adopted responses,^21^

Although scientists do their best to respond, dispel and publish proper information in reputable high-quality medical journals, the reliability of some of the information was sometimes questioned, with some articles being retracted soon after publication. This highlights the need to assure high ethical standards for scientific research during Pandemics. The general population has become more self-educated and empowered with access to information. In order to regain people's expectations, by providing transparent and accurate medical information, the scientific community must adhere to high-quality research standards. It should establish research networks with skilled knowledgeable experts who are capable to guide with integrity the world during this difficult period. In cases of global emergencies, there is an added duty to maintain universally accepted standards of ethics. The WHO has repeatedly issued guidelines and recommendations calling for the adaptation of high ethical standards during the current COVID-19 Pandemic 22. We are continuously learning during this difficult period. The most important lesson we should carry is not to forget these lessons, even those that are coming.
